# Distinct sub-clusters of developmental disorder-associated variants in the switch II region of RAC1

**DOI:** 10.1038/s41431-026-02105-x

**Published:** 2026-05-12

**Authors:** Hebah O. Althebaiti, James Cooksedge, Martin J. Baker, Madeleine Smith, Verity Edwards-Scott, André Luiz Santos Pessoa, Henri Margot, Kay A. Metcalfe, Mélanie Fradin, Hailey Mostow, Marielle Alders, Phillis Lakeman, Angeliki Malliri, Siddharth Banka, Tom H. Millard

**Affiliations:** 1 Division of Developmental Biology and Medicine, Faculty of Biology, Medicine and Health, University of Manchester, Manchester, UK; 2 Department of Biological Sciences, College of Science, University of Jeddah, Jeddah, Saudi Arabia; 3 Division of Cancer Science, Manchester Cancer Research Centre, University of Manchester, Manchester, UK; 4 Federal University of Ceara, Fortaleza, Brazil; 5 Service de Génétique Médicale, CHU de Bordeaux, Bordeaux, France; 6 Manchester Centre for Genomic Medicine, Manchester Academic Health Science Centre, University of Manchester, St Mary’s Hospital, Manchester, UK; 7 Service de Génétique Clinique, CLAD Ouest, CHU Rennes, Rennes, France; 8 St. Christopher’s Hospital for Children, Philadelphia, PA USA; 9 Amsterdam UMC, Department of Human Genetics, University of Amsterdam, Amsterdam, The Netherlands; 10 Reproduction and Development Research Institute, Amsterdam, The Netherlands; 11 Division of Evolution, Infection and Genomics, School of Biological Sciences, Faculty of Biology, Medicine and Health, University of Manchester, Manchester, UK

**Keywords:** Diseases of the nervous system, Neurodevelopmental disorders, Neurodevelopmental disorders

## Abstract

RAC1 is a signal transducer essential for neurodevelopment. Missense variants in *RAC1* cause heterogeneous neurodevelopmental disorders whose features include intellectual disability and developmental delay. Individuals with *RAC1* variants are categorized by head circumference into microcephalic, normocephalic and macrocephalic groups. We previously described a cohort of individuals with variants affecting the N-terminal part (Q61-R68) of switch II, a functional domain of RAC1. This cohort was normocephalic and their variants increased RAC1 signalling activity. Here, we report 15 new individuals with variants in switch II of RAC1. We describe clinical features for two individuals with variants affecting the N-terminal part of switch II (Q61-R68) and four individuals with variants in its C-terminal part (P69-Q74). All individuals exhibit intellectual disabilities and neuroradiological anomalies. Consistent with our previous study, individuals with variants in the N-terminal part of switch II were normocephalic. By contrast, individuals with variants in the C-terminal part of switch II exhibited microcephaly. Cell-based assays revealed that N- and C-terminal variants affect RAC1 function differently: N-terminal variants are activating, while C-terminal variants are dominant-negative. In a *Drosophila* model, we observe divergent effects on neuronal morphology, with switch II N-terminal variants increasing the complexity of dendritic arbors, while switch II C-terminal variants reduce dendritic complexity. Switch II N-terminal variants increase locomotor activity of adult *Drosophila*, while C-terminal variants had minimal effect, suggesting diverging effects on neuronal function and behaviour. We conclude that variants affecting N- and C-terminal parts of RAC1 switch II cause phenotypically and mechanistically distinct disorders.

## Introduction

RAC1 is a highly conserved Rho family GTPase that functions as a signal transducer in many cellular processes [1]. It acts as a molecular switch, cycling between an inactive GDP-bound form and an active GTP-bound form that binds to and regulates effector proteins. RAC1 is regulated by guanine nucleotide exchange factors (GEFs), which accelerate exchange of GDP for GTP, thus activating RAC1; GTPase-activating proteins (GAPs), which inactivate RAC1 by accelerating GTP hydrolysis and guanine-nucleotide-dissociation inhibitors (GDIs), which sequester GDP-bound RAC1 [2]. GTP/GDP bind to RAC1 via conserved motifs called G domains G1-5, while effectors and regulators interact via switch I (residues 26-45) and switch II (residues 59-74) regions, which undergo conformational changes during GDP/GTP exchange [3]. RAC1 regulates many cellular processes such as cell movement, proliferation and apoptosis [4]. Effectors of RAC1 include the WAVE regulatory complex (WRC), through which RAC1 promotes formation of lamellipodia and cell spreading and the protein kinase PAK [5–7]. RAC1 is involved in neurodevelopmental processes, including regulation of axon and dendrite growth, synapse assembly and function and neuronal survival, proliferation and migration [8, 9].

Previously, we reported that heterozygous *RAC1* missense variants cause a group of heterogeneous neurodevelopmental disorders (RAC1-NDD, OMIM #617751) characterised by variable combinations of neurological phenotypes (including developmental delay (DD), intellectual disability (ID), hypotonia, seizures and behavioural problems), neuroradiological features (such as cerebellar hypoplasia, corpus callosum hypoplasia, enlarged ventricles and polymicrogyria) and extra-neurologic features (such as craniofacial dysmorphism and cardiac abnormalities) [10]. Individuals displayed a broad spectrum of head circumferences, ranging from extreme microcephaly to extreme macrocephaly. We found that some *RAC1* variants associated with microcephaly had dominant negative (DN) effects on RAC1 function, leading us to propose that microcephaly may be a consequence of DN RAC1 variants [10].

More recently, we reported five pathogenic missense variants within the switch II region of RAC1 (Q61E, Y64D, Y64C, R68G and R68S) in eight individuals, mostly with head circumferences within the normal range [11]. These variants increased levels of active RAC1, promoted cell spreading and activated PAK and WRC effector pathways. This led us to propose that variants affecting Q61-R68 of the RAC1 switch II domain are activating and cause a distinct subclass of RAC1-NDD [11]. Pathogenic variants have not previously been reported in the C-terminal part of switch II (P69-Q74) of RAC1, with the exception of one individual with a P73L variant [10]. It is therefore not known if the phenotypic and mechanistic similarity of variants affecting the N-terminal part of switch II (Q61-R68) is a universal feature of variants anywhere in switch II.

We now describe 15 new individuals with eight variants affecting five sites within switch II of RAC1 and functionally analyse four variants. Three affect the C-terminal region (P69-Q74), allowing us to test whether variants in this region have similar effects to those in its N-terminal region (Q61-R68). Unlike individuals with Q61-R68 variants, who are normocephalic, individuals with L70-P73 variants exhibit microcephaly. Functional analysis of these L70-P73 variants suggests they have a DN effect on RAC1 function. Therefore, pathogenic variants affecting L70-P73 in the C-terminal section of switch II of RAC1 are phenotypically and mechanistically distinct from those affecting the N-terminal section of switch II.

## Materials and methods

### Identification and classification of switch II *RAC1* variants

Missense variants in the switch II encoding region of *RAC1* were collected via personal collaborations and the ClinVar database (https://www.ncbi.nlm.nih.gov/clinvar/) [12]. The clinical significance of variants was determined based on the American College of Medical Genetics and Genomics (ACMG) and the Association for Molecular Pathology (AMP) guidelines [13], using the InterVar tool (https://wintervar.wglab.org/). Variants follow HGVS nomenclature using RefSeq references with accession and version numbers (RAC1 MANE-Select NM_006908.4, protein NP_008839.2) encoding the disease-relevant shorter RAC1 isoform and the GRCh38 genomic reference (NC_000007.14). Annotation was performed using the VariantValidator tool (https://variantvalidator.org/) [14]. Informed consent from participants or their legal representatives was obtained in all cases.

### Mammalian expression plasmids

R66G, L70S, S71F and P73L mutations of human *RAC1* were generated using the QuikChange Lightning (Agilent Technologies) according to the manufacturer’s protocol, then subcloned into pRK5-myc. RAC1-WT, -T17N, Q61L and -R68G in pRK5-myc have been described previously [11]. TIAM1-C1199 pFlagCMV was obtained from Angeliki Maliri (University of Manchester), RAC1pRaichu [15] was obtained from Prof. Patrick Caswell (University of Manchester) and *RAC1* variant versions of this plasmid were generated using QuikChange Lightning. DHPH1-TRIO in pEGFPC1 was obtained from Addgene and subcloned into pRK5myc. Plasmids were transfected using FUGENE or FUGENE HD (Promega).

### Drosophila lines

GFPpUASTattB was generated by subcloning the eGFP coding region into pUASTattB. Variants of *Drosophila RAC1* were generated using QuikChange Lightning, then subcloned into GFPpUASTattB. Transgenic flies were generated by phiC31-mediated insertion into the attP40 integration site by the Manchester Fly Facility. For expression in type IV sensory neurons, these lines were crossed to ppk-Gal4 (Bloomington 32079) recombined with UAS-CD8-mCherry (Bloomington 27392). For pan-neuronal expression, these lines were crossed with elav-Gal4 (Bloomington 8765).

### Cell spreading and immunofluorescence

NIH3T3 fibroblast spreading assays were performed as previously described [11], except that the spreading period was 45 min rather than 30 min. Antibodies: Rabbit anti-myc (Proteintech 16286-1-AP); Anti-FLAG (Proteintech 66008-4-Ig); Alexa-568 anti-rabbit (Invitrogen A-21206); Alexa-488 anti-mouse (Invitrogen A-21202). F-actin stained with Alexa-405-phalloidin (Invitrogen A30104).

### FRET-based PAK1 binding assays

HEK293T cells were seeded in 24-well plates and 24 h later, transfected with RAC1-variant-pRaichu plasmids + TRIO-DHPHpRK5myc. After 24 h, cells were rinsed gently with cold PBS, then suspended in 1 ml PBS and transferred to cuvettes. Cuvettes containing the suspended cells were placed in a FluoroMax-4 spectrofluorometer (Horiba Scientific), excited at 433 nm and the emission intensities from 450 nm to 550 nm were recorded in 1 nm increments. FRET efficiency was quantified by dividing the peak YFP (523-525 nm) emission intensity by the peak CFP (473-475 nm) emission intensity. Statistical analysis was performed using GraphPad Prism. Data was statistically analysed using the Shapiro-Wilk test for distribution and one-way ANOVA with Tukey’s multiple comparison test.

### GST-pulldown PAK1 binding assays

HEK293 cells were seeded into 6 cm plates and 24 h later, transfected with RAC1 variants in pRK5-myc. After 18 h, active RAC1 was isolated following the protocol of Baker et al. [16]. Samples were run on 12% Bis-Tris polyacrylamide gels and transferred onto PVDF membrane. Membranes were probed for Myc-tag and alpha-tubulin (Cell Signaling Technologies, #2276 and #2144, respectively). Mouse and rabbit IgG-specific antibodies conjugated to HRP were used to detect bands and imaged with chemiluminescence on a BioRad Chemidoc.

### Cell circularity analysis

Images were acquired using a Leica DM6000B microscope using a 63x oil objective and Prime BSI Express CMOS camera. Anti myc-RAC1 and anti-FLAG-Tiam1 labelling was used to detect transfected cells. All image analysis was performed using ImageJ. The periphery of transfected cells was determined on the phalloidin channel using the threshold function in ImageJ-Fiji, then the cell circularity index (4π × area/perimeter^2^) was quantified using the ‘analyze particles’ function. Statistical analysis was performed using GraphPad Prism. Data was statistically analyzed using the Shapiro-Wilk test for distribution and the Kruskal-Wallis test with Dunn’s Test for difference.

### Imaging *Drosophila* type IV sensory neurons

Wandering third instar *Drosophila* larvae were washed briefly with water, then mounted dorsal-side up in 80% glycerol. A coverslip was placed over the larvae and pressed to flatten larva and remove internal organs. Images of UAS-CD8-mCherry channel of dorsal class IV sensory neurons of segments A1-A4 were collected using a Nikon A1R confocal microscope, 20× Plan-Apo dry objective. Images were analysed and processed using ImageJ-Fiji as follows: neurons were isolated using the ‘clear outside’ function, rotated so that the dorsal midline was at the top of the image and then thresholded. Sholl analysis was performed on the dorsal hemishell of the neuron centred on the cell body.

### Locomotor activity in *Drosophila*

Male flies 5–7 post-eclosion were placed in polycarbonate tubes containing fly food at one end and sealed with cotton wool at the other. The vials were placed in a Trikinetics *Drosophila* activity monitor (DAM) system at 25 °C with a 12/12-h light/dark cycle. Fly movement was monitored using infrared beams positioned along the tube. Each passage of a fly through a beam was recorded as a ‘move’. Data collection commenced 48 h after placing flies in the DAM system to allow recovery from CO_2_ anaesthesia and acclimatisation to the light/dark cycle. Data was processed using DAMFileScan and statistically analysed by Kruskal-Wallis and Dunn’s multiple comparisons tests using Graphpad Prism software

### Structural modelling

Models of RAC1 variants bound to Tiam1 DH domain and RhoGDI were generated using AlphaFold2 accessed via Colabfold [17] using the UCSF ChimeraX interface [18]. All models are closely aligned with published crystal structures of RAC/Tiam1 or RAC1/RhoGDI complexes. Affinities were estimated using the PRODIGY web server [19]. Images were generated using ChimeraX.

## Results

### The spectrum of *RAC1* switch II variants is wider than previously recognised

**Fig. 1:**
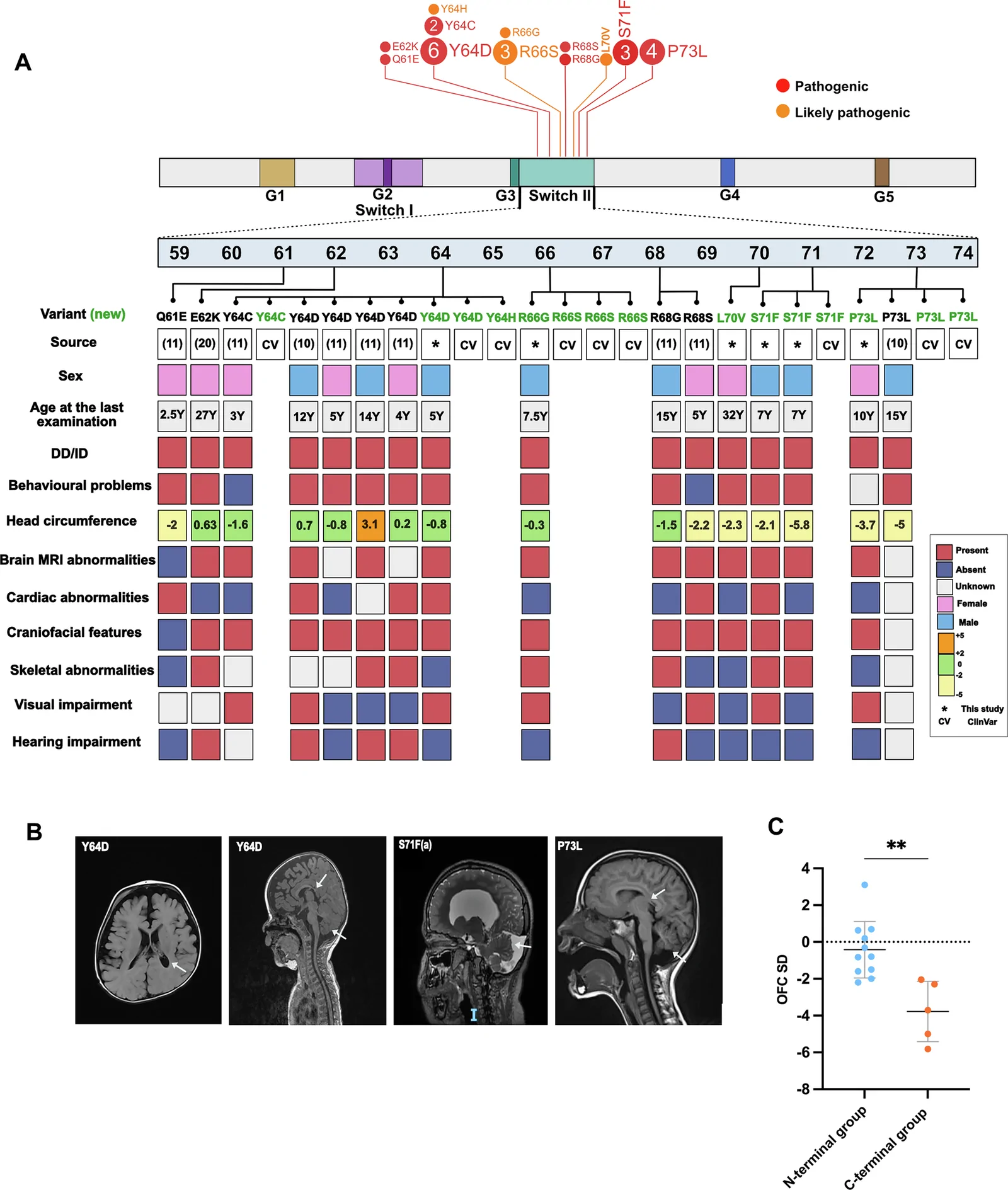
Expansion of genotype and phenotype spectrum of *RAC1* variants in the switch II region. **A** A schematic of missense variants within switch II of RAC1. The missense *RAC1* variants were collected from different sources (personal collaborations, ClinVar). The variants were classified pathogenic (red) or likely pathogenic (orange). The number within the circle indicates the number of individuals affected with this variant. Schematic of RAC1 illustrates the location of the guanine nucleotide-binding domains (G domains, G1-G5) and switch I and II regions. Variants present in newly identified individuals are coloured green in the ‘Variants’ row. The ‘Source’ row indicates whether individuals are previously published, newly described in this study (*) or listed on ClinVar (CV), but clinical details are unavailable. The diagram includes the pathogenic RAC1 switch II variants and their corresponding main phenotypes. Additionally, the sex of individuals, the source of the variants and head circumference. **B** Brain MRI for the individuals with indicated *RAC1* variants. The first two scans from the left showed the brain scan for Y64D, showing plagiocephaly and a dilated lateral ventricle. cerebellar atrophy, thin posterior body of the corpus callosum. The scan for the S71F and P73L individuals showed cerebellar hypoplasia with a thin corpus callosum. Key features indicated by arrows. **C** Graph showing standard deviation (SD) of occipitofrontal circumference (OFC) for individuals with N-terminal and C-terminal switch II variants. Data normality was confirmed using the Shapiro–Wilk test. Statistical analysis was conducted with an unpaired Welch’s t-test (*t* = 3.860, *df* = 7.354, two-tailed, *α* = 0.05), comparing OFC SD values between the N-terminal group (*n* = 11, mean = –0.4194) and the C-terminal group (*n* = 5, mean = –3.775). The mean difference was 3.355 ± 0.8693 (95% CI: 1.319–5.391, R² = 0.6695), indicating a significant effect (*P* = 0.0057). Variance between groups did not differ significantly (F₄,₁₀ = 1.141, *P* = 0.7832). Individuals with N-terminal variants are shown as blue points and those with C-terminal variants as orange points.

Firstly, we set out to examine the genotype spectrum of *RAC1* switch II variants. Through collaborations and variant databases, we identified 15 individuals with eight distinct variants, who have not been previously reported in the medical literature (Fig. 1A). Six of these individuals had previously reported variants (Y64C, Y64D and P73L) [10, 11]. The remaining nine individuals have novel variants (Y64H, R66G, R66S, L70V, S71F). All the novel variants were classed as either pathogenic (S71F) or likely pathogenic (Y64H, R66G, R66S and L70V) according to ACMG/AMP guidelines [13]. Combining these data with the 10 previously reported individuals means we are now aware of 12 distinct switch II variants (at the amino acid level) in *RAC1* affecting 8 out of 16 residues (Fig. 1A). Y64 is the most frequently substituted residue in the N-terminal part of switch II, while P73L is the most recurrent variant in its C-terminal section (Fig. 1A). These data substantially expand the known *RAC1* switch II variant spectrum.

### *RAC1* switch II N- and C-terminal variants associate with partially distinct phenotypes

Next, where possible, we accessed clinical data of newly identified affected individuals and compared them with that of the previously reported individuals with *RAC1* variants within switch II [10, 11, 20]. Detailed clinical data were available for six newly identified individuals, two with switch II N-terminal region variants (Y64D and R66G) and four with switch II C-terminal region variants (L70V, S71F and P73L). All six individuals were reported to have DD and ID varying from mild to severe (Fig. 1A and Supplementary Table 1). Behavioural problems such as autism, hyperactivity, hypersociability and difficulty managing emotions were reported in five individuals. Delayed independent walking and speech development were observed in most individuals. Head circumferences ranged from –0.3 to –5.8 SD. Neuroradiological anomalies were reported in all six individuals, mainly affecting the corpus callosum and cerebellum (Fig. 1B). Thinning of the corpus callosum was observed in three individuals with Y64D, R66G and P73L, while partial or complete agenesis of the corpus callosum was noted in two individuals with the S71F variant (Supplementary Table 1). Additionally, cerebellar abnormalities were present in four individuals: Y64D, L70V, S71S and P73L. Craniofacial dysmorphology was present in all individuals, while cardiovascular defects were noted in two.

Next, we examined phenotypic differences between individuals with variants in N- and C-terminal regions of switch II. This analysis included the new individuals described here and previously reported individuals (Fig. 1A–C and Supplementary Table 1). All individuals with switch II variants exhibited DD and ID. The majority also showed behavioural problems, brain MRI abnormalities and craniofacial features, while some had cardiac, skeletal, visual and hearing impairments. However, a remarkable divergence in occipitofrontal circumference (OFC) was observed between individuals with N- and C-terminal variants. OFC was within the range –2 to 3.1 SD for N-terminal variants, with most individuals exhibiting normal OFC, while for C-terminal variants, the OFC range was –5.8 to –2 SD, with all individuals exhibiting microcephaly. This difference in head circumference between the two groups was statistically significant (Fig. 1C). Collectively, these data indicate that the phenotype of switch II variants is characterised by DD, ID and brain abnormalities; there is a marked divergence in head circumference between individuals with variants in the N- and C-terminal regions of switch II.

### Variants affecting the N-and C-terminal regions of switch II affect RAC1 signalling activity differently

Previous functional analysis of RAC1 switch II variants revealed that variants affecting the N-terminal part of switch II (Q61-R68) activate RAC1. However, the impact of the variants in the C-terminal part of switch II (P69-Q74) on RAC1 activity remains unknown. This was of particular interest because of our observation of distinct phenotypes from C- and N-terminal variants. We therefore aimed to determine the effect of three novel switch II variants (R66G, L70V, S71F) on RAC1 activity and P73L, for which previous functional studies were inconclusive [10].

**Fig. 2:**
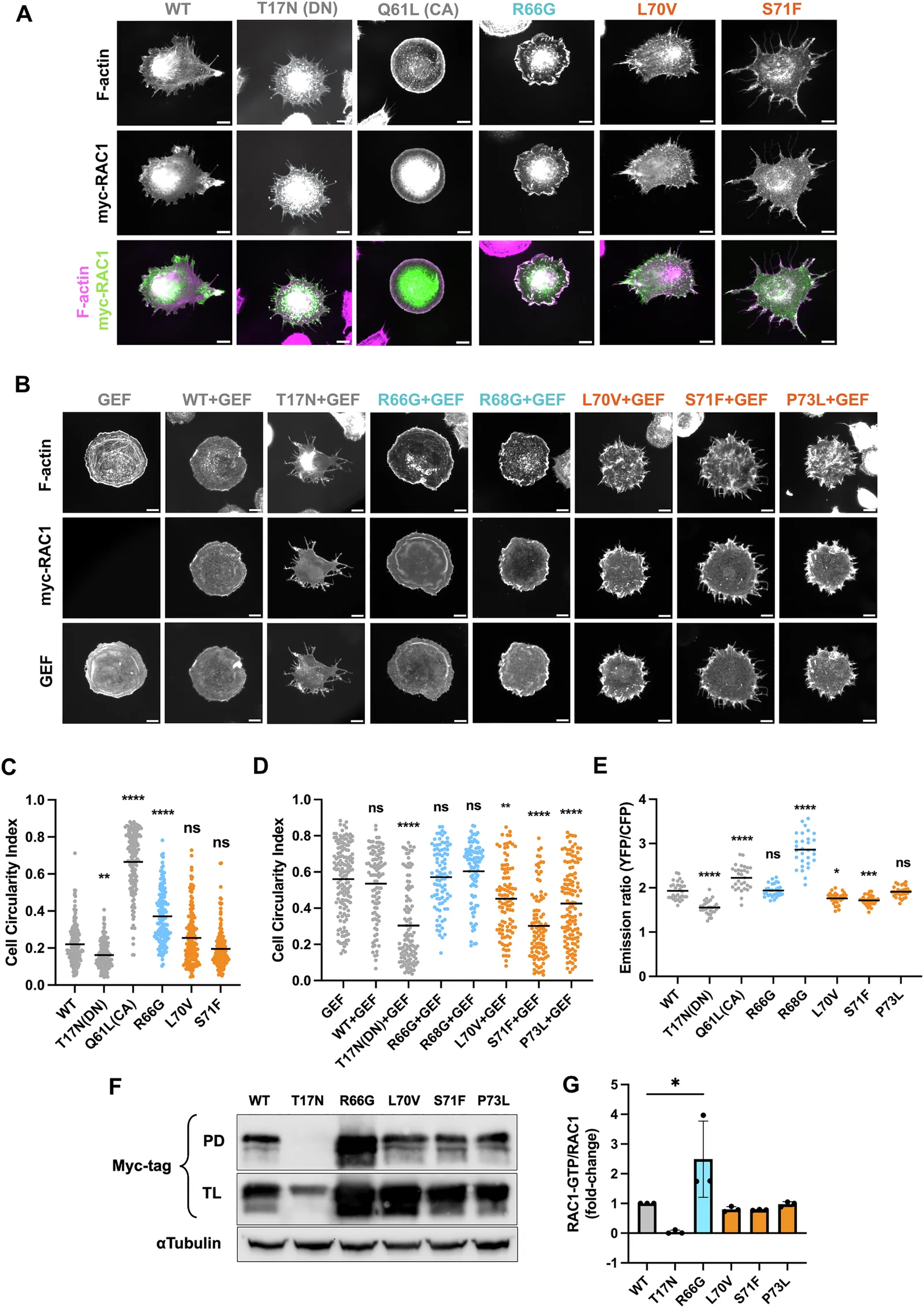
Differential effects of N- and C-terminal switch II variants on cellular RAC1 activity. **A** Representative images of NIH 3T3 fibroblasts transfected with the indicated RAC1 variants illustrate the most common morphology for each variant. Cells stained with anti-myc, labelling transfected cells (green) and Alexa405-Phalloidin to label F- actin (magenta). **B** Images of NIH 3T3 fibroblasts transfected with indicated RAC1 variants and the GEF Tiam1. Cells stained with anti-myc to label cells transfected with RAC1 variants, anti-flag to label cells transfected with the GEF Tiam1 and Alexa405-Phalloidin to label F- actin. All scale bars indicate 10 μm. **C** Graph shows cell circularity values of individual transfected cells, with lines indicating group means. Each point represents one cell. *n* > 140 cells from 9 coverslips. Statistical analysis was performed using the Kruskal-Wallis test (*H* = 466.5, *df* = 5, *P* < 0.0001), followed by Dunn’s multiple comparisons test (α = 0.05). Comparisons to WT, the *P*-values: T17N (*P* = 0.0019), Q61L (*P* < 0.0001), R66G (*P* < 0.0001), L70V (*P* > 0.9999) and S71F (*P* = 0.8749). Controls are shown in grey, activating variants in blue and dominant-negative variants in orange. **D** Graphs show the cell circularity values of individual transfected cells and the line indicates the mean value. Statistical analysis was performed using the Kruskal-Wallis test (*H* = 200.5, *df* = 7, *P* < 0.0001), followed by Dunn’s multiple comparisons test (α = 0.05). Comparisons to GEF alone, *P*-values: WT + GEF, R66G + GEF and R68G + GEF (*P* > 0.9999), T17N + GEF (*P* < 0.0001), L70V + GEF (*P* = 0.0037), S71F + GEF (*P* < 0.0001) and P73L + GEF (*P* < 0.0001),. *n* > 90 cells from 9 coverslips. Controls shown in grey, activating variants in blue and dominant negative variants in orange. **E** Graph showing emission ratio of YFP to CFP for populations of HEK293 cells expressing RAC1 Raichu biosensor modified to include the indicated variant. *n* = 30 cell populations. Statistical analyses were performed using one-way ANOVA (*F*(7232) = 114.8, *P* < 0.0001). Comparisons to WT, the *P*-values: T17N (*P* < 0.0001), Q61L (*P* < 0.0001), R68G (*P* < 0.0001), L70V (*P* = 0.0376) and S71F (*P* = 0.0018), both R66G and P73L (*P* > 0.9999). Asterisks indicate the significant differences in the data from WT-RAC1 Raichu. **F** Representative GST-*P*AK1 pulldown assay blot showing total (TL) and pulled-down (PD) myc-RAC1. αTubulin is a loading control. **G** Quantitation of pulled-down RAC1/Total RAC1 from three independent experients and normalised to RAC1-WT. Statistically analysed using one-way ANOVA. (∗*p* < 0.05, ∗∗*p* < 0.01, ∗∗∗*p* < 0.001, ∗∗∗∗*p* < 0.0001.

RAC1 promotes lamellipodia formation during fibroblast spreading on an adhesive surface [21]. Lamellipodia formation is inhibited by the known DN RAC1 mutant T17N, resulting in a stellate morphology. By contrast, the constitutively active (CA) RAC1 mutant Q61L increases lamellipodia formation, resulting in a circular cell morphology (Fig. 2A–C). Circularity of spreading fibroblasts therefore offers a simple readout of RAC1 activity at the cellular level. Expression of the new variant R66G in fibroblasts promoted lamellipodia formation and a significant increase in cell circularity index (Fig. 2), suggesting that, like other Q61-R68 variants, R66G increases RAC1 activity. Expression of the C-terminal switch II variants L70V and S71F did not significantly alter the cell circularity compared to the WT-RAC1. This aligns with our previous findings for the P73L variant [10]. These results suggest that variants affecting L70-P73 may affect RAC1 activity differently from those affecting Q61-R68.

### Switch II C-terminal variants inhibit GEF-induced cell spreading

Since variants affecting the C-terminal part of switch II do not activate RAC1, we hypothesized they may have DN effects. Although no change in cell circularity was observed on expressing these variants in fibroblasts, the reduction in circularity caused by the strong DN T17N was small; weaker DN effects may not be detectable with this assay. To increase the sensitivity of the assay, we co-expressed an activated form of the RAC1 GEF Tiam1 to increase endogenous RAC1 activity (Fig. 2B, D). Expressing the GEF on its own resulted in increased lamellipodia formation and circularity. Co-expression of WT-RAC1 with the GEF did not change cell circularity compared to expression of GEF alone; co-expressing the DN variant T17N caused a pronounced decrease in circularity (Fig. 2B, D). Co-expressing the activating switch II variants R66G and R68G with the GEF did not change circularity compared to GEF alone. By contrast, co-expressing variants in the C-terminal part of switch II (L70V, S71F and P73L) significantly decreased circularity and inhibited lamellipodia formation, with cells instead exhibiting filopodia at the cell periphery and apical actin patches (Fig. 2B, D). This suggests that the C-terminal *RAC1* switch II variants inhibit GEF-induced cell spreading and therefore likely act as DNs, albeit more weakly than the strong DN variant T17N.

### Switch II C-terminal variants reduce RAC1 signalling activity

The RAC1 effector PAK1 binds specifically to the active GTP-bound RAC1 and RAC1 binding to the CDC42 and RAC interactive binding domain (CRIB) of PAK1 is commonly used as an indicator of RAC1 activity. We investigated the impact of variants on RAC1/PAK1 binding, using the RAC1-Raichu biosensor [15], in which RAC1 binding to PAK1-CRIB increases the YFP: CFP emission ratio due to increased fluorescence resonance energy transfer (FRET) efficiency. HEK293T cells were co-transfected with the Raichu biosensor modified to contain RAC1 variants alongside an activated form of the GEF TRIO, which was used because it activates RAC1 more robustly than Tiam1 in this assay. The YFP: CFP ratio of transfected cell populations was measured using a fluorimeter (Fig. 2E). Cell populations expressing RAC1-CA-Raichu exhibited a significant increase in the YFP: CFP emission ratio, compared to RAC1-WT, indicating increased PAK1 binding, while the opposite effect was observed for RAC1-DN-Raichu (Fig. 2E). The activating variant R68G-Raichu exhibited an increased YFP/CFP ratio, but unexpectedly, cells expressing R66G-Raichu showed no change in YFP: CFP ratio relative to RAC1-WT. The C-terminal switch II variants L70V-Raichu and S71F-Raichu exhibited a significant decrease in YFP: CFP ratio, indicating reduced PAK1 binding (Fig. 2E). The mean YFP: CFP ratio for P73L-Raichu was slightly lower than that of WT-RAC1-Raichu; this difference was not statistically significant. These data suggest that the L70V and S71F variants reduce RAC1/PAK1 binding in the presence of an active GEF, consistent with the DN effects of these variants observed in cell spreading assays. To assess the effect of variants on PAK1 binding by an alternative method and in the absence of an activated GEF, we performed GST-PAK1 pulldown assays using lysates of HEK293 cells expressing *RAC1* variants. In this assay, significantly more RAC1-R66G was pulled down than RAC1-WT, consistent with the increased activity observed in spreading assays (Fig. 2F, G). Across three repeats, L70V and S71F consistently exhibited reduced PAK1 binding compared to RAC1-WT; variation in the extent of reduction meant that statistical significance was not reached. The level of PAK1 binding to P73L appeared similar to RAC1-WT in this assay (Fig. 2F, G). The results of the two PAK1 binding assays were therefore broadly consistent, with the exception of R66G, which increased PAK1 binding in the pulldown assay, but not in the FRET-based assay.

### N-terminal and C-terminal switch II variants have opposite effects on neuronal morphology in vivo

Previously, we observed that expressing the activating variant Y64D in class IV dendritic arborization (cIVda) neurons in *Drosophila* larvae increased dendrite branching and reduced the size of the dendritic arbor [11]. Having established that variants affecting L70-P73 of switch II have opposite effects on RAC1 activity to those affecting Q61-R68 in cell-based assays, we asked whether these variants also affected neuronal morphology differently in vivo. We therefore investigated the effects of one representative variant from each group, R68G and P73L, in our *Drosophila* cIVda neuron model.

**Fig. 3:**
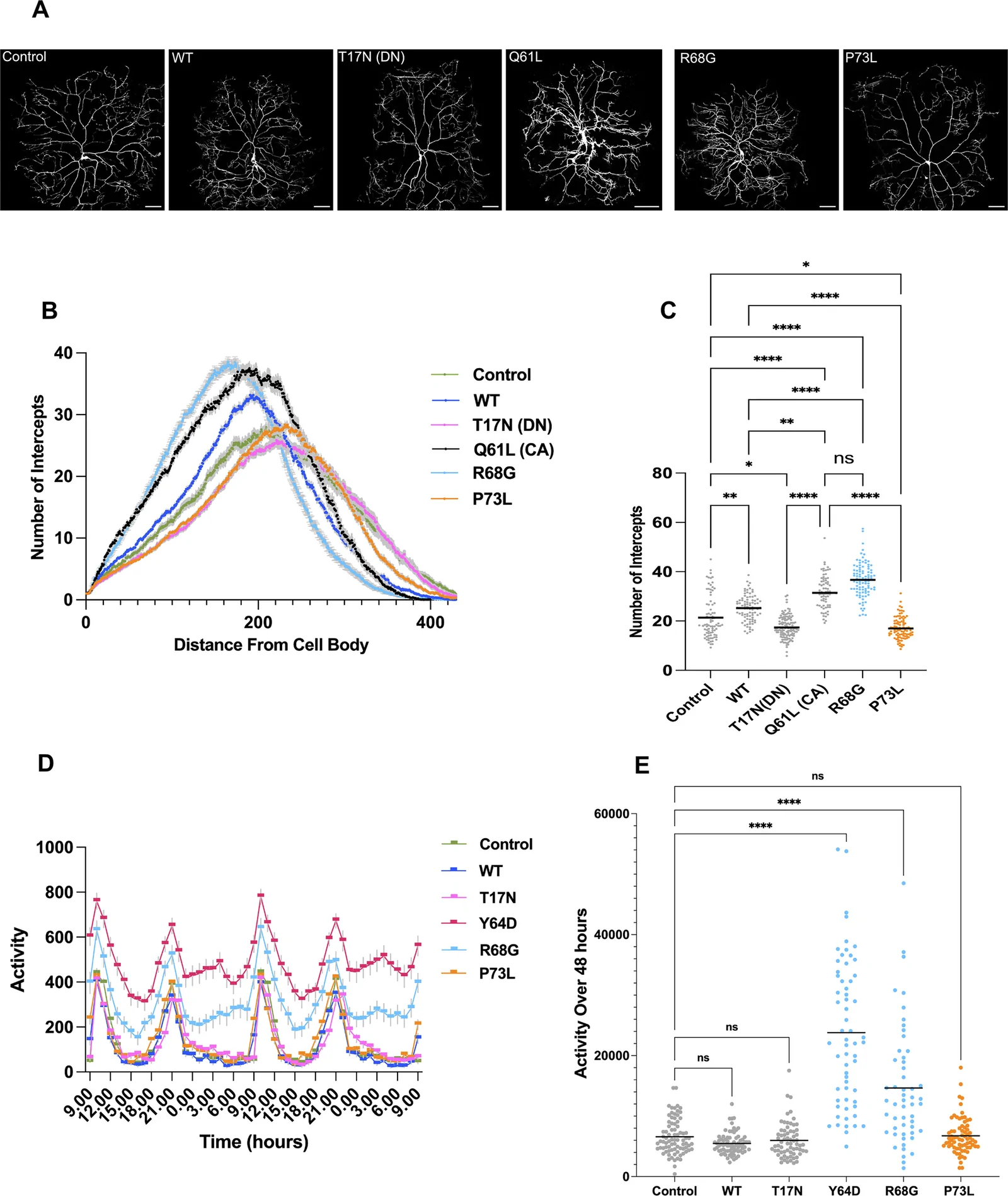
N-terminal and C-terminal switch II variants have different effects on Drosophila neuronal morphology and behaviour. **A** Representative images of neuronal morphology of cIVda sensory neurons from segments A1-A4 from the dorsal surface of third instar larvae of the indicated genotype. CD8-mCherry was used to visualise neurons expressed under the control of ppk-Gal4. Scale bars indicate 100 µm. **B** Sholl analysis of dendritic organisation. A series of semi-circles originating from the cell body is projected onto neuron images; the number of intercepts describes the number of times a dendrite intercepts a semi-circle at a certain radius from the cell body. The graph shows the mean ± SEM of *n* > 80 neurons for each data set. **C** Quantification of the number of intercepts at 150 μm from the cell body. Each dot represents an individual neuron, controls shown in grey, activating variants in blue and dominant negative variants in orange. Statistical analysis was performed using the Kruskal-Wallis test (H = 265.7, df = 4, *P* < 0.0001), followed by Dunn’s multiple comparisons test (*α* = 0.05). *P*-values for comparisons to control are: WT (*P* = 0.0003), T17N (*P* = 0.0451), R68G (*P* < 0.0001) and P73L (*P* = 0.0166). **D** Drosophila circadian locomotor activity. The graph shows the sum of mean ± SEM of movement events recorded in each 30-min period over 48 h for the indicated genotype. Flies were maintained in a 12/12-h light/dark cycle (light 9.00–21.00). *n* = >50 flies per genotype. **E** Plot shows total movement events for flies of the indicated genotype over 48 h. Each dot represents an individual fly, controls shown in grey, activating variants in blue and dominant negative variants in orange. *n* = >50 flies per genotype. Statistical analysis was performed using the Kruskal-Wallis test (H = 170.3, df = 5, *P* < 0.0001), followed by Dunn’s multiple comparisons test (α = 0.05). Adjusted *P*-values for comparisons to control were: Y64D (*P* < 0.0001), R68G (*P* < 0.0001), WT (*P* = 0.8289), P73L (*P* > 0.9999) and T17N (*P* > 0.9999). Asterisks indicate the significant differences (∗*p* < 0.05, ∗∗*p* < 0.01, ∗∗∗*p* < 0.001, ∗∗∗∗*p* < 0.0001) relative to control and WT.

As with RAC1-Y64D [11], expression of GFP-RAC1-R68G, or the CA control Q61L, increased the complexity and reduced size of dendritic arbors compared to GFP alone or GFP-RAC1-WT (Fig. 3A–C). Sholl analysis was used as a readout of the dendrite distribution within the arbor and this showed increased dendrite number close to the cell body, with a significantly increased number of interceptions at 150 µm from the cell body for GFP-RAC1-R68G compared to GFP-RAC1-WT (Fig. 3B, C). By contrast, GFP-RAC1-P73L exhibited reduced dendritic complexity close to the cell body, similar to the result obtained for the known DN mutant T17N (Fig. 3B, C).

These results suggest that N-terminal activating *RAC1* switch II variants increase dendrite branching, while a C-terminal DN *RAC1* switch II variant reduces dendrite branching, demonstrating opposing effects of these variants on neuronal morphogenesis in vivo.

### Activating switch II variants causes hyperactive locomotion of *Drosophila*

We examined whether altered neuronal morphology in *Drosophila* might correlate with altered neuronal function by assessing the effect of neuronal expression of *RAC1* variants on adult locomotor activity, a well-established *Drosophila* behavioural assay [22]. *Drosophila* exhibit predictable circadian variation in locomotor activity, with peaks at dawn and dusk and periods of sleep and reduced activity between. Locomotor activity was measured using a Trikinetics *Drosophila* Activity Monitor system. Expressing GFP-RAC1-WT in neurons did not significantly alter the total locomotor activity of flies compared to expression of GFP alone (Fig. 3D, E). By contrast, expressing the activating switch II variants Y64D or R68G significantly increased total activity. Y64D and R68G expressing flies still exhibited increased activity at dawn and dusk, however during periods between, in which activity is greatly reduced in control flies, activity remained high for Y64D and R68G expressing flies. Expression of the DN variant P73L did not significantly alter total movement or circadian variation in movement compared to flies expressing GFP alone. Expressing the DN mutant T17N also had no effect on activity; thus, in this assay, it appears that activating variants increase locomotor activity while DN variants have no observable effect.

These results suggest that differences in the molecular, cellular and developmental effects of N-terminal activating and C-terminal DN *RAC1* switch II variants ultimately result in different behavioural phenotypes.

### Structural analysis of switch II variants

**Fig. 4:**
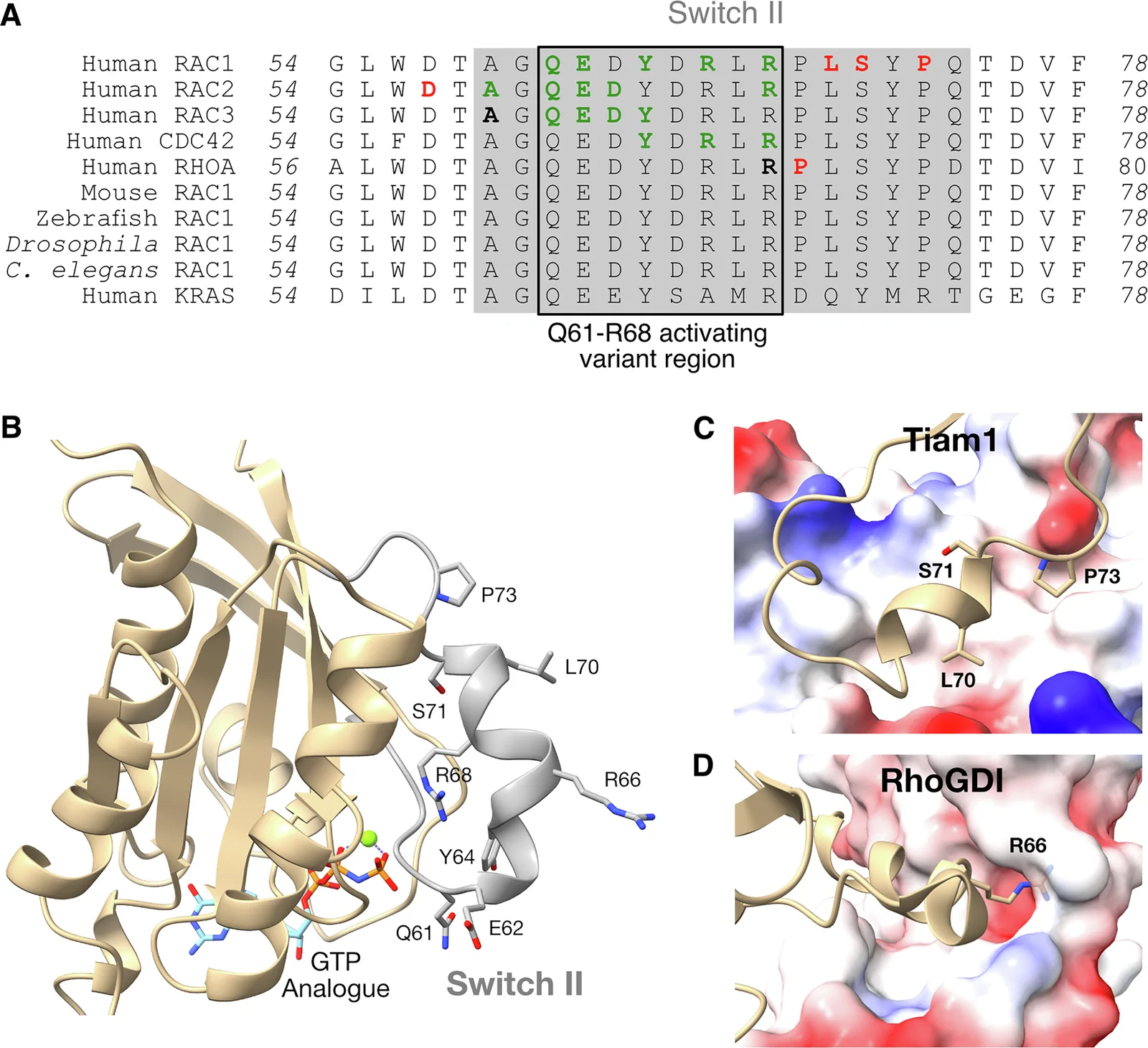
Structural analysis of pathogenic variants within switch II of RAC1. **A** Amino acid sequence of RAC1 switch II region aligned with and that of selected orthologs and related GTPases. Grey shading indicates the switch II region, while the black box indicates the activating N-terminal region of switch II (Q61-R68). Residues in bold are affected by published pathogenic variants in the indicated human Rho GTPase: green = activating, red = dominant negative, black = effect unknown [29–34]. **B** Crystal structure of RAC1 bound to GTP analogue (PDB ID 1MH1) with switch II residues affected by pathogenic variants indicated [35]. **C** Interface of RAC1 with Tiam1, with L70, S71 and P73 indicated (PDB ID 1FOE) [23]. **D** Interface of RAC1 with RhoGDI, with R66 indicated (PDB ID 1HH4) [24]. In C and D, RAC1 is shown in gold as a ribbon, while Tiam1 and RhoGDI are shown as surfaces (red: negative charge, blue: positive charge).

Next, we explored the structural basis of the opposing effect of variants in the N and C-terminal regions of switch II on RAC1 signalling activity. Firstly, we examined the conservation of switch II residues substituted in participants. Apart from R66, all variant-affected residues in the N-terminal activating region (Q61-R68) displayed conservation across both the Rho GTPase family and the wider RAS superfamily (Fig. 4A). By contrast, while the C-terminal region (P69-Q74) is conserved in other Rho GTPases, conservation across the wider RAS superfamily is low, for example none of the residues in this region are identical in human KRAS (Fig. 4A). This conservation pattern suggests that the C-terminal region is likely to be important for features shared between Rho family GTPases, such as interaction with Rho GEFs, GAPs and GDIs, but less significant for features common to all small GTPases, such as GTP binding and hydrolysis, or maintaining the protein fold (Fig. 4B). The side chains of newly identified variants (R66G, L70, S71 and P73) are solvent exposed, consistent with a role in interaction with other proteins, rather than intramolecular interactions within RAC1 and the distance from the nucleotide binding site and side chain orientation means a direct contribution to nucleotide binding or hydrolysis is unlikely (Fig. 4B). L70, S71 and P73 all form part of the binding interface between RAC1 and the GEF Tiam1 (Fig. 4C) [23], so substitution of any of these residues could alter the affinity of RAC1 for Tiam1 and related GEFs. To model the impact of the C-terminal variants on GEF binding, we used AlphaFold2 to predict the structure of each variant complexed with the DH domain of Tiam1 (Supplementary Fig. 1), then used the PRODIGY affinity prediction tool [19] to estimate the binding affinity for Tiam1 of each variant. All variants were predicted to modestly reduce binding affinity, with ΔΔG for the interaction estimated at 0.6 kcal/mol for L70V, 0.2 kcal/mol for S71F and 0.7 kcal/mol for P73L. R66 is a key element of the binding interface between RAC1 and RhoGDI [24], occupying a negatively-charged cavity in the surface of RhoGDI (Fig. 4D). Prediction of the effect of the R66G variant on the interaction between RAC1 and RhoGDI using the same approach as described for the C-terminal variants suggested that this variant reduces binding affinity of RAC1 for RhoGDI with an estimated ΔΔG of 0.9 kcal/mol (Supplementary Fig. 1).

## Discussion

Ten individuals with RAC1 switch II variants have previously been reported, all but one affecting the Q61-R68 sub-region of switch II. These individuals presented with DD/ID, cardiac abnormalities and craniofacial features; most had a head circumference within the normal range and functional analyses indicated that these Q61-R68 variants activate RAC1 [11, 18]. In this study, we describe 15 further individuals with switch II variants. Based on the available clinical data, individuals with Y64D and R66G variants exhibited features consistent with those of previous individuals with variants in the Q61-R68 region, including normocephaly. However, the new individuals with variants outside this region (L70V, S71F and P73L) exhibited microcephaly or severe microcephaly, suggesting a different disease mechanism.

Our functional analysis demonstrated that expression of the R66G variant, within the Q61-R68 region, enhanced fibroblast spreading, consistent with our existing hypothesis that variants in this region increase RAC1 activity [11]. Previous structural studies of the RAC1-RhoGDI complex revealed that R66 is important in the interaction of RAC1 with RhoGDI and a substitution at this site disrupts this interaction, providing a possible mechanism for the activating effect of this variant [24, 25]. We observed an increase in PAK1 binding for R66G in our pulldown assay, but not using the Raichu biosensor. This biosensor is known to be insensitive to the effects of RhoGDI on RAC1 activity, so the discrepancy between these two PAK1 binding assays is consistent with the hypothesis that R66G activates by disrupting the RAC1/RhoGDI interaction [15].

Functional analyses of variants in the C-terminal part of switch II (L70V, S71F, P73L) suggest that these variants have the opposite effect on RAC1 signalling activity to those in the N-terminal part (Q61-R68). Expression of RAC1-L70V, -S71F or -P73L reduced the ability of the GEF Tiam1 to induce cell spreading, suggesting DN effects. Consistent with this, RAC1-L70V and -S71F both exhibited reduced binding to PAK1 compared to RAC1-WT. RAC1-P73L did not reduce PAK1 binding, which could indicate that this variant has pathway-specific effects or that any effect on PAK1 binding is too small to detect using this assay. What is the molecular basis for the DN effects of these variants? L70, S71 and P73 have all been implicated in GEF binding and notably, substitution of L70 and S71 for alanine has been found to abolish the ability of GEFs to promote nucleotide exchange on RAC1, without preventing the RAC1/GEF interaction [23, 26]. L70V, S71F and P73L variants may therefore act as DNs by competing with WT RAC1 for GEF binding but not undergo activation on GEF binding and therefore not stimulate downstream effector pathways. The discrepancies in the strength of dominant negative effects that we observed in different assays may suggest these variants are somewhat selective in terms of which pathways and processes they affect. Why might the N- and C-terminal switch II variants exhibit opposite effects on RAC1 activity? The N-terminal part of switch II overlaps with the nucleotide-binding G3 box, so variants in this region may directly alter the intrinsic GTP/GDP cycle of RAC1, for example, accelerating nucleotide cycling or slowing GTP hydrolysis. Q61 plays a direct role in GTP hydrolysis and substitution of this residue causes constitutive activation throughout the small RAS superfamily [27]. R68 forms several intramolecular hydrogen bonds [11], so it may be important in maintaining the structure of G3 and switch II. Y64 lies adjacent to the nucleotide binding site and forms part of the interaction surface for several RAC1 regulators and effectors, so the effects of its substitution may be complex [11, 28]. The C-terminal part of switch II is more distant from the nucleotide binding site, so variants in this region are more likely to alter signalling activity by disrupting interactions with regulators or effectors rather than by directly impacting nucleotide cycling.

Previously, we observed that expression of the activating variant Y64D in *Drosophila* increased dendritic arbor complexity and reduced arbor size in cIVda neurons [11]. Here, we expressed a different activating switch II variant, R68G and observed a similar increase in arbor complexity and reduction in size to that observed for Y64D. This further supports our hypothesis that variants affecting Q61-R68 have similar effects on RAC1 functions in neurodevelopment. Interestingly, expression of the DN variant P73L in cIVda neurons had the opposite effect to activating variants, reducing dendritic complexity. A similar effect was observed on expression of the strong DN variant T17N, thus providing evidence that P73L acts as a DN in vivo, in a neurodevelopmental setting. Overall, these findings support the hypothesis that N-terminal and C-terminal switch II variants have activating and DN effects, respectively.

Many individuals with RAC1 switch II variants exhibit behavioural issues, including sleep difficulties, hyperactivity and repetitive behaviours. To examine whether activating and DN variants might affect behaviour differently, we examined the effects of RAC1-Y64D, -R68G and -P73L on locomotor activity in *Drosophila*. Flies expressing the activating variants Y64D and R68G exhibited significantly increased activity, particularly during periods when control flies typically sleep. However, flies expressing the DN variant P73L or the control DN T17N did not significantly affect activity. These results support the notion that activating and DN switch II variants have different effects on neuronal function in vivo. Hyperactivity and altered sleep patterns are observed in some individuals with activating switch II RAC1 variants [11], so it is interesting that these behaviours are also observed in flies. RAC1 contributes to many developmental processes that could underlie this hyperactivity phenotype, including regulation of axon and dendrite growth and guidance and synapse formation and function. Further work using this *Drosophila* model has the potential to reveal mechanisms underlying behavioural disorders in RAC1-NDD individuals.

In conclusion, our study reveals that *RAC1* switch II variants fall into two distinct groups: activating variants affecting Q61-R68, typically associated with normal head circumference and DN variants affecting L70-P73, associated with microcephaly. Within these two groups, there is considerable phenotypic variation between individuals, suggesting that other factors besides whether the variant is activating or DN influence the disease phenotype, and therefore, considerably more work is needed to understand the specific effect of individual variants. As more individuals and variants are described, patterns are emerging and this will ultimately lead to improved diagnosis and management of RAC1-NDDs.

## Supplementary information

Supplementary Figure 1

Supplementary Table 1

Supplementary material legends

## Data Availability

Experimental data will be shared on request.
