# Supplementary material for: Distinct sub-clusters of developmental disorder-associated variants in the switch II region of RAC1
Source: Eur J Hum Genet. 2026 May 12;34(11):1719–28. doi: 10.1038/s41431-026-02105-x (PMC13743744; doi:10.1038/s41431-026-02105-x)
Supplement: Supplementary file 1 [file 41431_2026_2105_MOESM3_ESM.docx]

**Supplementary table 1. Clinical features of individuals with RAC1 switch II variants**

Individuals 1-11 have variants within N-terminal region of switch II (patient # green), while individuals 12-16 have variants in the C-terminal region of switch II (patient # red).

**Supplementary figure 1. Alphafold2 models of RAC1 switch II variants bound to Tiam1 and RhoGDI**. AlphaFold2 models closely match crystal structures of RAC1/Tiam1 and RAC1/RhoGDI complexes shown in Figure 4. Tiam1 and RhoGDI are shown as surfaces (red: negative charge, blue: positive charge) while the switch II region of RAC1 is shown as a ribbon. **(A)**. Models of interface of switch II region of RAC1-WT, -L70V, -S71F and -P73L with Tiam1. L70 forms hydrophobic interactions within a non-polar cleft in the surface of Tiam1, which the smaller side chain of valine only partially occupies. The S71F substitution brings hydrophobic phenylalanine into proximity with polar glutamine 163 of Tiam1, while the P73L substitution brings leucine close to proline 125 of Tiam1. **(B)** Models of interface of switch II region of RAC1-WT and R66G with RhoGDI. Positively charged R66 occupies a negatively charged cavity in RhoGDI, an interaction eliminated by the R66G substitution.
